# Clinical relevance of *TP53* hotspot mutations in high-grade serous ovarian cancers

**DOI:** 10.1038/s41416-019-0654-8

**Published:** 2019-11-29

**Authors:** Musaffe Tuna, Zhenlin Ju, Kosuke Yoshihara, Christopher I. Amos, Janos L. Tanyi, Gordon B. Mills

**Affiliations:** 1 Department of Medicine, Baylor College of Medicine, Houston, TX USA; 2 Precision Oncology, Knight Cancer Institute, Oregon Health & Science University, Portland, OR USA; 3 Department of Bioinformatics and Computational Biology, The University of Texas MD Anderson Cancer Center, Houston, TX USA; 4 Department of Obstetrics and Gynecology, Niigata University, Niigata, Japan; 5 Department of Obstetrics and Gynecology, University of Pennsylvania, Philadelphia, PA 19104 USA; 6 Department of Systems Biology, The University of Texas MD Anderson Cancer Center, Houston, TX USA; 7 Department of Cell, Developmental & Cancer Biology, Oregon Health & Science University, Portland, OR USA

**Keywords:** Prognostic markers, Ovarian cancer

## Abstract

**Background:**

Mutation of *TP53* is the most frequent genetic alteration in high-grade serous ovarian cancer (HGSOC). The impact of hotspot mutations of *TP53* and protein levels on patient outcomes in HGSOC has not been fully elucidated.

**Methods:**

The study population (*n* = 791) comprised of HGSOC samples with *TP53* mutation from TCGA and other publicly available data. Univariate and multivariate cox proportional hazards regression analyses were used to select variables that were correlated with patient survival.

**Results:**

We assessed the effects of *TP53* mutations based on type and individual hotspot mutations on patient outcomes in HGSOC. Only hotspot mutations were associated with outcomes. Three hotspot mutations: G266, Y163C, and R282, in aggregate were associated with a worsened overall and recurrence-free survival compared with other hotspot mutations (*p* < 0.0001 and *p* = 0.001), other non-hotspot missense mutations (*p* < 0.0001 and *p* = 0.008), truncated mutations (*p* < 0.0001 and *p* = 0.001), and all other mutations (*p* < 0.0001 and *p* = 0.001). Specific hotspot mutations were associated with different protein expression patterns consistent with different functions.

**Conclusions:**

This study provides evidence that individual *TP53* hotspot mutations have different impact on HGSOC patient outcomes and potentially TP53 function. Thus the status of particular *TP53* aberrations could influence response to therapy and selection of therapeutic agents.

## Background

Ovarian cancer is the fifth most common cancer among women with over 21,290 and 225,500 new cases diagnosed and 14,180 and 140,200 death estimated annually in the United States and the world, respectively.^1,2^ *TP53* is the most frequently mutated tumour-suppressor gene in human cancer with the highest frequency in high-grade serous ovarian cancer (HGSOC; at least 96%).^3^ Some *TP53* mutations result in loss of wild-type functions either by loss of DNA-binding activity or by a dominant-negative effect whereby the mutated allele inhibits function of the wild-type allele.^4^ However, some mutations appear to provide a gain-of-function independent of wild-type *TP53*.^5^ Gain-of-function mutations can increase cell transformation and contribute to chemotherapy resistance.^6,7^ In addition, functional consequences of *TP53* mutations may depend on the specific mutation or on the type of mutations. For example, frameshift mutations have been proposed to cause a different phenotype than missense mutations.^8^ A number of *TP53* missense mutations produce full-length p53 proteins that frequently have a prolonged half-life with accumulation of inactive protein, whereas frameshift mutations do not usually lead to accumulation of p53, and nonsense mutations generally result in an unstable protein.^9^ *TP53* mutations have been classified by their location [such as DNA-binding domain (DBD); the most common site of aberrations], oncogenic function [gain-of-function (GoF) or loss-of-function (LoF)], and by type of mutation (missense, nonsense, frameshift, splice site, and indel). Missense mutations have also been subclassified into structural (suspected effect on their protein structure and activity) and functional classes (based on their capacity to *trans*-activate promoters of p53 target genes).^10^ Moreover, five TP53 mutations were classified as temperature sensitive. Nevertheless, each mutation has different features.^11^ For instance, different amino acid substitutions at the same site can have different functions: R248Q mutation enhances in vitro invasiveness in lung cancer cell lines, while R248W cannot increase invasiveness in human NCI-H1299 cell lines.^12^ Similarly, R273H and R273C enhance cell proliferation, invasion, and drug resistance in vitro, but R273G does not.^13^ Perhaps due to the complexity of different classes, studies assessing the association of p53 status and clinical outcome have frequently produced conflicting results.^14–18^

Multiple studies have evaluated the clinical relevance of *TP53* mutations in HGSOC; however, they have yielded inconsistent results. This can be due to *TP53* mutation status being inferred by incomplete sequencing or the assessment method of *TP53* mutations being inadequate.^19^ Further including different histologic subtypes or grades of ovarian cancer combined with lack of robust clinical trial grade outcomes data can influence the conclusions. For example, some but not all missense mutations in *TP53* lead to accumulation of inactive protein (80% correlation), while frameshift and nonsense mutations do not lead to accumulation of p53.^9^ Thus immunohistochemical staining is insufficient to identify samples with *TP53* mutations. In addition, initial *TP53* sequencing focused on exons 5–8. However, many *TP53* mutations occur outside this region. Furthermore, the type or location of a specific mutation can alter the functional outcomes. Little is known about the effects of specific mutations in*TP53* on patient outcome in HGSOC. Therefore, in this study we evaluated the prognostic significance of hotspot mutations, different types of *TP53* mutations, and association with levels of specific proteins as an indication of functional consequences.

## Methods

### Subjects

The Cancer Genome Atlas (TCGA) generated *TP53* mutations and clinical data were retrieved from XENA (https://genome-cancer.ucsc.edu).^3^ Normalised RPPA data was consolidated from MD Anderson Cancer Center TCPA data portal (http://app1.bioinformatics.mdanderson.org/tcpa/_design/basic/index.html). Overall survival (OS) time was counted from the date of diagnosis of HGSOC to the date of death or last follow-up. Recurrence-free survival (RFS) time was counted from the date of diagnosis of ovarian cancer to the date of recurrence or last follow-up. Sample and clinical data were based on a March 2018 freeze from TCGA data portal. TCGA mutations data was used as training set. In addition, we recruited publicly available *TP53* mutation and clinical data to develop a validation set.^14,20–29^ Demographic characteristics are summarised in Supplementary Table S1. In this study, we included only primary HGSOCs with *TP53* mutation. Samples without mutation were excluded. In the training set, 468 samples were included with *TP53* mutations. Two samples were excluded, owing to these two samples harbouring tow hotspot mutations: R273 and R248. In the validation set, 325 samples were included with *TP53* mutation. In total, 791samples (466 from TCGA and 325 from validation sets) were included in this study. Six samples from the training set, and 18 samples from validation set were excluded from survival analysis due to missing clinical data. This study followed REMARK (reporting recommendations for tumour-marker prognostic studies) criteria.

### Statistical analysis

Chi-square and Wilcoxon rank-sum analysis were performed to identify differences between groups. Univariate Cox proportional hazards regression analysis was used to select variables that were correlated with RFS time and/or OS time. Kaplan–Meier survival curves were drawn with RFS and OS probabilities for groups with and without *TP53* hotspot mutations and between hotspot mutations and type of mutations. Log-rank test was used to determine whether RFS and OS probability were significantly different between the groups. Multivariate cox proportional hazard regression model was used to select independent prognostic variables. Benjamini–Hochberg method was used to display the survival differences between patient groups.^30^ To identify proteins significantly expressed between groups, we applied Student’s *t* test. The *p* value < 0.05 was considered significant. Statistical analyses were performed using R 2.14.0 (www.r-project.org) and STATA (www.stata.com).

## Results

### Patterns of *TP53* mutation in HGSOC

*TP53* mutations are essentially universal in HGSOC. This requires a unique approach to identify associations with outcomes. Rather than comparing the effects of different *TP53* mutations between mutant and non-mutant tumours, it is necessary to compare effects of different mutation types or locations on patient characteristics. Further, this requires evaluation of the effects of specific types of mutations in large sample sets. We thus classified mutations in HGSOC by mutation type (frameshift, splice site, nonsense, and in frame) or hotspot locations in two independent sample sets as well as a combined data set to increase power.

The most frequent types of mutation in *TP53* in HGSOC were missense mutations (60.52%), followed by frameshift (15.24%), splice site (10.52%), nonsense (10.73%), and in-frame mutations (3.22%).^3^ A total of 126 (44.68%, 126/282) hotspot mutations was observed in HGSOC in TCGA data (training set), with the nine most common hotspot mutations being: R273 (20.63%, 26/126), R248 (16.67%, 21/126), R175 (14.29%, 18/126), Y220 (9.52%, 12/126), I195 (9.52%, 12/126), C176 (8.73%, 11/126%), G245 (8.73, 11/126), S241 (6.35%, 8/126), and Y163 (6.35%, 8/126). No significant difference was observed in the patterns of hotspot mutations between younger age (patients with age ≤55 years) and older age (patients with age >55 years) (*p* = 0.637) and also between early stage (I and II) and late stage (III and IV) (*p* = 0.563). R248 mutations were observed only in late-stage patients (*p* = 0.090) but limited numbers precluded a statistically significant correlation. Similarly, the type of mutation was not significantly different between patients with younger and older age (*p* = 0.416) and patients with early and late stages (*p* = 0.402) (Supplementary Table S2).

In the validation set, only five hotspot mutations were identified: R273, R248, R175, Y220, and G245. Of note, in the validation set frameshift mutations were higher in patients with younger age (≤55 years) compared with patients with older age (>55 years) (*p* = 0.031, *q* = 0.155), while no difference was observed between patients with younger and older age in missense, nonsense, splice site, and in-frame mutations (*p* = 0.184). In general frequency of hotspot mutations was similar between younger and older patients (*p* = 0.071). G245 was observed only in older patients (*p* = 0.081) or late stage (*p* = 0.207), whereas R248 was seen mostly in patients with older age (*p* = 0.139), and R175 was observed more often in patients with younger age (*p* = 0.027). No significant difference was found in frequency of type of mutations and hotspots between early and late stage (*p* = 0.899 and *p* = 0.454, respectively).

To increase statistical power, we combined data sets. Total 19 hotspot mutations were found in the combined set. We found no significant difference between younger and older age and between early and late stage in type of mutation (*p* = 0.283 and *p* = 0.770, respectively) and in hotspot mutations (*p* = 0.407 and 0.991, respectively). V157F was observed only in late-stage patients (*p* = 0.350). In general, results were relatively consistent between the training and validation set.

### Association of *TP53* mutations with survival in HGSOC

We tested whether different types of *TP53* mutations are associated with survival in the training set (TCGA data set). In univariate analysis, no association was found between cases with different type of mutations (e.g., missense vs nonsense, missense vs splice site, missense vs frameshift, missense vs in frame, nonsense vs frameshift, nonsense vs splice site, nonsense vs in frame, frameshift vs splice site, frameshift vs in frame, and splice site vs in frame) and OS or RFS (Supplementary Table S3). Truncated mutations, including nonsense, frameshift, and splice site mutations, are predicted to cause LoF. Thus we compared missense mutations with truncated as well as in-frame mutations. We did not find significant difference in survival between the truncated mutations and other type of mutations (Supplementary Table S3).

We then tested association of hotspot mutations with 5 years of survival time. In univariate analysis, R273 mutations were associated with better OS than R248, Y163C, G266, and R282 (*p* = 0.013, *p* = 0.003, *p* = 0.006, *p* = 0.013, respectively). However, no difference was found between mutations at R273 and R175, C176, I195, Y220, C238, S241, G245, C275, and P278 on OS (Table 1). R273 mutations were associated with better RFS than patients with R282 (*p* = 0.007). Older age and late stage were associated with reduced OS (*p* < 0.0001 and *p* = 0.006, respectively), while only stage was associated with shorter RFS (*p* = 0.001) (Tables 1 and 2).

**Table 1:** Univariate 5 years of overall survival analysis of *TP53* hotspot mutations in HGSOC.

Covariates	Training set (TCGA)				Validation set				Combined data			
	HR	*p*	*q*	95% CI	HR	*p*	*q*	95% CI	HR	*p*	*q*	95% CI
R273 vs Y220	1.16	0.314	0.461	0.87–1.56	1.11	0.482	0.675	0.83–1.48	1.14	0.220	0.372	0.93–1.39
R273 vs S241	1.03	0.715	0.867	0.89–1.19					0.98	0.701	0.907	0.87–1.10
R273 vs G245	1.03	0.749	0.867	0.85–1.24	0.96	0.613	0.693	0.80–1.14	0.99	0.837	0.969	0.87–1.12
R273 vs I195	1.10	0.397	0.546	0.88–1.39					1.11	0.267	0.420	0.92–1.35
R273 vs C176	0.99	0.938	0.977	0.81–1.22					0.99	0.960	0.998	0.85–1.17
R273 vs R175	0.90	0.651	0.842	0.59–1.40	1.16	0.474	0.675	0.77–1.75	0.99	0.998	0.998	0.74–1.35
R273 vs H193	0.95	0.272	0.427	0.86–1.04					0.99	0.570	0.799	0.91–1.05
R273 vs R248	2.86	**0.013**	**0.026**	1.25–6.55	1.21	0.652	0.693	0.52–2.81	1.81	**0.039**	0.072	1.03–3.16
R273 vs C238	1.09	0.125	0.212	0.98–1.23	0.96	0.383	0.670	0.88–1.05	0.99	0.801	0.970	0.93–1.06
R273 vs C275	1.00	0.977	0.977	0.92–1.10	1.02	0.652	0.693	0.94–1.11	1.00	0.951	0.998	0.94––1.07
R273 vs P278	0.99	0.824	0.906	0.87–1.12	1.10	0.189	0.378	0.96–1.25	1.03	0.581	0.799	0.94–1.12
R273 vs Y163C	1.29	**0.003**	**0.009**	1.09–1.52					1.25	**<0.0001**	**<0.0001**	1.11–1.39
R273 vs G266	1.13	**0.006**	**0.015**	1.04–1.24					1.14	**<0.0001**	**<0.0001**	1.06–1.22
R273 vs R282	1.10	**0.013**	**0.026**	1.02–1.19					1.08	**0.007**	**0.014**	1.02–1.15
R273 vs Y163C/G266/R282	5.25	**<0.0001**	**<0.0001**	2.18–12.63	4.56	**0.005**	**0.014**	1.57–13.21	4.71	**<0.0001**	**<0.0001**	2.44–9.10
Group 2 vs Group 1	0.18	**<0.0001**	**<0.0001**	0.10–0.34	0.25	**0.001**	**0.004**	0.11–0.56	0.21	**<0.0001**	**<0.0001**	0.13–0.34
Group 2 vs non-hotspot miss mut	0.29	**<0.0001**	**<0.0001**	0.16–0.52	0.36	**0.013**	**0.030**	0.16–0.81	0.57	**<0.0001**	**<0.0001**	0.45–0.72
Group 2 vs all other mut	0.25	**<0.0001**	**<0.0001**	0.14–0.45	0.26	**0.001**	**0.004**	0.12–0.57	0.53	**<0.0001**	**<0.0001**	0.42–0.66
Group 2 vs truncated	0.24	**<0.0001**	**<0.0001**	0.13–0.43	0.26	**0.001**	**0.004**	0.12–0.58	0.26	**<0.0001**	**<0.0001**	0.16–0.41
Group 2 vs in frame	0.39	**0.031**	0.057	0.17–0.92					0.26	**0.001**	**0.002**	0.11–0.59
Age ≤55 vs >55 years	1.61	**<0.0001**	**<0.0001**	1.25–2.08	0.94	0.693	0.693	0.71–1.26	1.31	**0.005**	**0.011**	1.09–1.59
Stage I and II vs Stage III and IV	2.70	**0.006**	**0.015**	1.34–5.46	2.49	**<0.0001**	**<0.0001**	1.51–4.11	2.48	**<0.0001**	**<0.0001**	1.64–3.74

miss; missense, mut; mutations, bold indicates statistically significant *p* values

**Table 2:** Univariate 5 years of recurrence-free survival analysis of *TP53* hotspot mutations in HGSOC.

Covariates	TCGA				Validation				Combined			
	HR	*p*	*q*	95% CI	HR	*p*	*q*	95% CI	HR	*p*	*q*	95% CI
R273 vs Y220	1.20	0.204	0.408	0.90–1.61	0.76	0.108	0.212	0.54–1.06	0.98	0.830	0.913	0.80–1.20
R273 vs S241	1.03	0.670	0.765	0.90–1.17					0.97	0.536	0.694	0.87–1.07
R273 vs G245	1.02	0.834	0.834	0.86–1.21	0.87	0.120	0.212	0.74–1.04	0.95	0.412	0.567	0.85–1.07
R273 vs I195	1.17	0.148	0.362	0.95–1.45					1.08	0.403	0.567	0.91–1.28
R273 vs C176	0.95	0.571	0.737	0.79–1.14					0.92	0.248	0.495	0.79–1.06
R273 vs R175	0.92	0.695	0.765	0.62–1.38	0.82	0.382	0.535	0.52–1.28	0.86	0.307	0.519	0.64–1.15
R273 vs H193	1.02	0.573	0.737	0.95–1.10					1.04	0.270	0.495	0.97–1.10
R273 vs R248	1.53	0.311	0.526	0.67–3.46	0.8	0.570	0.606	0.36–1.75	1.14	0.635	0.776	0.66–1.98
R273 vs C238	1.02	0.759	0.795	0.92–1.12	1.02	0.535	0.606	0.96–1.09	1.03	0.330	0.519	0.97–1.08
R273 vs C275	1.02	0.603	0.737	0.95–1.10	0.95	0.305	0.474	0.87–1.04	0.99	0.714	0.827	0.94–1.05
R273 vs P278	1.03	0.578	0.737	0.92–1.16	0.96	0.606	0.606	0.83–1.12	1.00	0.921	0.921	0.92–1.10
R273 vs Y163C	1.11	0.185	0.407	0.95–1.31					1.11	0.102	0.249	0.98–1.25
R273 vs G266	1.05	0.280	0.513	0.96–1.16					1.07	0.119	0.262	0.98–1.15
R273 vs R282	1.11	**0.007**	**0.040**	1.03–1.20					1.06	**0.016**	**0.044**	1.01–1.12
R273 vs Y163C/G266/R282	2.62	**0.023**	0.063	1.14–6.00	2.38	0.094	0.212	0.86–6.57	2.43	**0.005**	**0.018**	1.31–4.53
Group 2 vs Group 1	0.43	**0.009**	**0.040**	0.23–0.81	0.43	**0.050**	0.212	0.18–0.99	0.41	**0.001**	**0.006**	0.25–0.68
Group 2 vs non-hotspot missense mut	0.45	**0.017**	0.053	0.23–0.87	0.51	0.121	0.212	0.21–1.20	0.70	**0.008**	**0.025**	0.54–0.91
Group 2 vs all other mut	0.41	**0.006**	**0.040**	0.22–0.77	0.46	0.067	0.212	0.20–1.06	0.67	**0.001**	**0.006**	0.52–0.86
Group 2 vs truncated	0.43	**0.008**	**0.040**	0.23–0.80	0.46	0.071	0.212	0.20–1.07	0.44	**0.001**	**0.006**	0.260.72
Group 2 vs in frame	0.28	**0.014**	0.051	0.10–0.78					0.29	**0.004**	**0.018**	0.12–0.67
Age ≤55 vs >55 years	1.09	0.468	0.735	0.86–1.38	0.91	0.541	0.606	0.69–1.22	1.01	0.874	0.916	0.85–1.22
Stage I and II vs Stage III and IV	2.81	**0.001**	**0.022**	1.54–5.13	5.79	**<0.0001**	**<0.0001**	3.06–10.95	4.00	**<0.0001**	**<0.0001**	2.59–6.21

Bold indicates statistically significant *p* values

In the validation set, no association was found between type of mutations and OS and RFS, except nonsense mutations. Nonsense mutations were associated with worse OS than splice site mutations (*p* = 0.023). Similar to the training set, no difference was found between mutations at R273 and R175, Y220, G245, and I195 on OS in the validation set. Sample size was too small to test survival effects of mutations at S241, I195, C176, Y163C, G266, and R282 in the validation set. Then we tested stage and age for association with survival. We found that late stage was associated with reduced OS (*p* < 0.0001) and RFS (*p* < 0.0001) (Tables 1 and 2). In general, the results between the training and validation sets were consistent, except the difference in OS between R273 and R248. This could be due to distribution of R248 mutations between stages; all R248-mutated samples were in late-stage tumours in the training set, while 23.08% of R248-mutated samples in the validation set were in early-stage tumours.

**Fig. 1:**
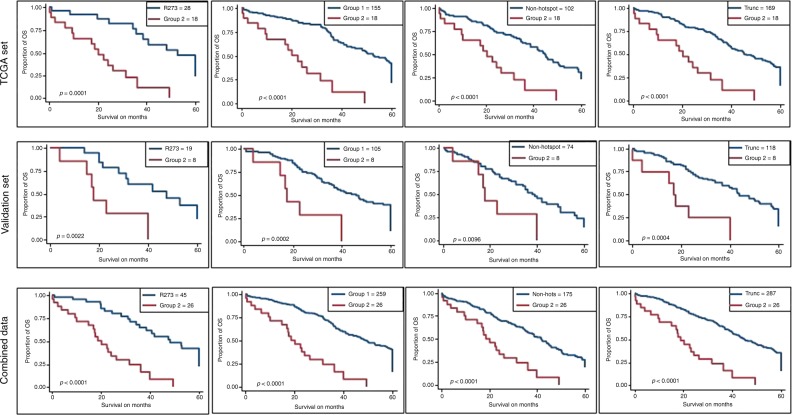
Overall survival and recurrence-free survival analyses. Kaplan–Meier plot of overall survival probability for patients with *TP53* hotspot mutations.

**Fig. 2:**
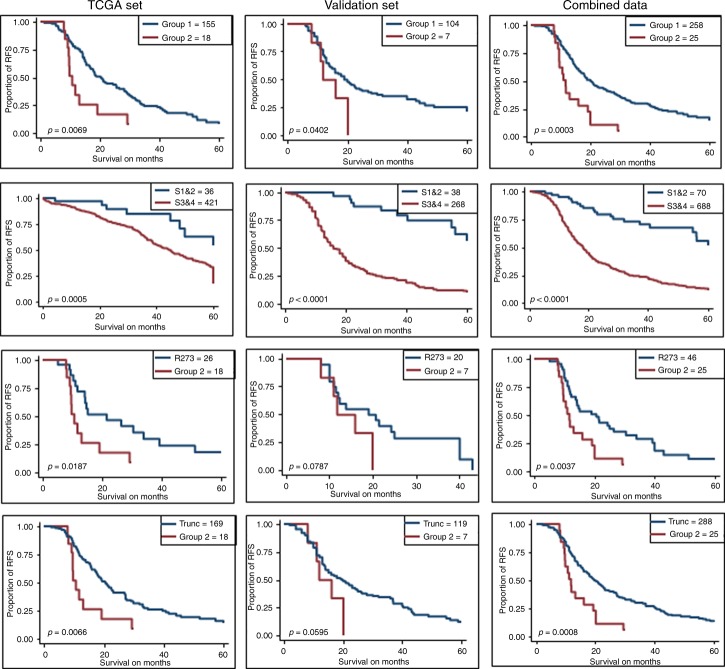
Kaplan–Meier plot of recurrence-free survival probability for patients with *TP53* hotspot mutations.

Next, we combined two sets to increase the sample size (*n* = 791). In the combined set, type of mutations was not associated with OS and RFS. Next, we identified 19 hotspot mutations of *TP53*: Y163, R175, C176, H179, H193, I195, Y220, Y234, C238, S241, G245, R248, G266, R273, C275, P278, D281, R282, and V157. We found no significant difference on OS between R248W and R248Q (*p* = 0.467). In contrast, OS was significantly different between Y163C and Y163N/H (*p* = 0.008). Based on Kaplan–Meier survival analysis, we identified two groups of hotspot mutations (Supplementary Fig. S1). Fifteen hotspot mutations: R175, C176, H179, H193, I195, Y220, Y234, C238, S241, G245, R248, R273, C275, P278, and D281, were considered as group 1, and three hotspots: G266, Y163C, and R282, were identified as group 2. V157 mutations were overlapping with both groups in Kaplan–Meier plot, therefore we did not include in either group. Group 1 hotspot mutations as a set were associated with better OS (*p* < 0.0001) and RFS (*p* < 0.001) than the group 2 mutations (Tables 1 and 2, and Figs. 1 and 2). We thus designated group 1 as good prognostic hotspot mutations and group 2 as poor prognostic hotspot mutations. Furthermore, group 2 was associated with a worsened OS and RFS than non-hotspot missense mutations (*p* < 0.0001 and *p* = 0.008, respectively), truncated mutations (*p* < 0.0001 and *p* = 0.001, respectively), (Tables 1 and 2, and Figs. 1 and 2), all other mutations (*p* < 0.0001 and *p* = 0.001, respectively), and in-frame mutations (*p* = 0.001 and *p* = 0.004, respectively) (Tables 1 and 2). When we tested each individual hotspot mutations, all good prognostic hotspot mutations were associated with a better OS than Y163C (Supplementary Table S4). All good prognostic hotspot mutations except I195 and P278 were associated with better OS than G266, while R273, R175, C176, S241, Y220, G245, D281, and C238 were associated with better OS than R282 (Supplementary Table S4). Patients with R273 mutations had better OS (*p* < 0.0001) and RFS (*p* = 0.005) than the patients with group 2 mutations. Early stage and younger age were associated with better OS (*p* < 0.0001 and *p* = 0.005, respectively; Fig. 1).

Then we tested the same hotspot mutations as groups in the training and validation sets. We found that patients with group 1 mutations had better OS (*p* < 0.0001) and RFS (*p* = 0.009) than the patients with group 2 mutations in the training set. A similar result was observed in the validation set, patients with group 1 mutations had prolonged OS (*p* = 0.001) and RFS (*p* = 0.05) compared to patients with group 2 mutations (Tables 1 and 2). Patients with group 2 mutations had worse OS in the training (*p* < 0.0001) and the validation set (*p* = 0.013) compared to patients with non-hotspot missense mutations. Besides, group 2 mutations were associated with shorten OS than all other mutations in the training set (*p* < 0.0001) and the validation set (*p* = 0.001) (Table 1 and Fig. 1). Indeed, group 2 mutations were associated with worse OS than truncated mutations in the training (*p* < 0.0001) and the validation (*p* = 0.001) sets (Table 1 and Fig. 1). Moreover, R273 mutations were associated with better OS than group 2 mutations in the training (*p* < 0.0001) and the validation (*p* = 0.005) sets (Fig. 1).

In multivariate analysis, group 1 hotspot mutations and younger age were associated with better OS (*p* < 0.0001, *p* = 0.009, respectively), while group 1 hotspot mutations were associated with prolonged RFS (*p* = 0.010) in the training set. Similarly, group 1 hotspot mutations were associated with prolonged OS (*p* = 0.001), and early stage (I and II) was associated with better RFS (*p* = 0.001) in the validation set. Furthermore, group 1 hotspot mutations, early stage, and younger age were associated with better OS (*p* < 0.0001, *p* = 0.025, and *p* = 0.005, respectively) in the combined set. Of note, group 1 hotspot mutations and early stage were associated with prolonged RFS (*p* = 0.002 and *p* < 0.0001, respectively) in the combined data set (Table 3). Moreover, we tested whether any correlation exist between type of mutations and *BRCA1* and *BRCA2* mutations. However, we found no correlation between *BRCA1* and *BRCA2* mutations and missense (*p* = 0.9338 and *p* = 0.8915, respectively), nonsense (*p* = 0.9635 and *p* = 0.9206, respectively), frameshift (*p* = 0.2957 and *p* = 0.9785, respectively), splice site (*p* = 4044 and *p* = 0.9562, respectively), and in-frame (*p* = 0.5313 and *p* = 8090, respectively) mutations of *TP53*.

**Table 3:** Multivariate analysis of OS and RFS in the training, validation, and combined sets.

Covariates	OS				RFS			
	HR	*p*	*q*	95% CI	HR	*p*	*q*	95% CI
Training set								
Group 1 vs Group 2	5.36	**<0.0001**	**<0.0001**	2.93–9.80	2.33	**0.010**	**0.022**	1.23–4.41
Stage I and II vs III and IV	2.78	0.153	0.172	0.68–11.33	1.67	0.264	0.396	0.68–4.11
Age ≤55 vs >55 years	1.78	**0.009**	**0.016**	1.16–2.74	0.90	0.592	0.592	0.62–1.32
Validation set								
Group 1 vs Group 2	3.88	**0.001**	**0.003**	1.71–8.82	1.85	0.164	0.295	0.78–4.38
Stage I and II vs III and IV	2.08	0.069	0.089	0.95–4.57	9.98	**0.001**	**0.005**	2.42–41.22
Age ≤55 vs >55 years	1.26	0.369	0.369	0.76–2.10	0.85	0.557	0.592	0.50–1.46
Combined set								
Group 1 vs Group 2	4.76	**<0.0001**	**<0.0001**	2.95–7.67	2.25	**0.002**	**0.006**	1.35–3.73
Stage I and II vs III and IV	2.26	**0.025**	**0.038**	1.11–4.60	3.96	**<0.0001**	**<0.0001**	1.86–8.46
Age ≤55 vs >55 years	1.59	**0.005**	**0.011**	1.15–2.20	0.90	0.497	0.592	0.67–1.22

Bold indicates statistically significant *p* values

Our analysis provides strong evidence that each hotspot mutation needs to be evaluated individually or in discrete sets to determine association with outcome rather than collectively. Larger data sets will help to identify the remaining mutations whether they have impact on survival.

### Protein expression is associated with different types of *TP53* mutation

Next, we assessed differentially expressed proteins between *TP53* hotspot mutations. Amounts of p53 (*p* = 0.014), ERα (*p* = 0.049), INPP4B (*p* = 0.001), VEGFR2 (*p* = 0.017), MSH2 (*p* = 0.020), and MSH6 (*p* = 0.012) were significantly higher in R248-mutated samples than in R175-mutated tumours, while CIAP (*p* = 0.039), PDK1 (*p* = 0.018), SF2 (*p* = 0.016), and Tuberin pT1462 (*p* = 0.015) were significantly higher in samples with R175 mutation than with R248 mutation (Supplementary Table S5). Expression of c-Jun pS73 (*p* = 0.024), myosinIIA pS1943 (*p* = 0.030), NDRG1 pT346 (*p* = 0.030), and NRF2 (*p* = 0.039) protein were significantly higher in R248-mutated samples compared with samples with R273 mutation. In contrast, protein expression of ASNS (*p* = 0.034), N-cadherin (*p* = 0.027), PCNA (*p* = 0.023), RAD51 (*p* = 0.027), BCL2A1 (*p* = 0.037), and PYGL (*p* = 0.034) were higher in R273-mutated samples compared with that in R248-mutated samples (Supplementary Table S5). We found a series of total and phosphorylated proteins that were significantly higher in samples with Y163C mutations compared to samples with group 1 mutations: cell cycle proteins (e.g., Cyclin B1), MEK/mitogen-activated protein kinase (MAPK) pathway proteins (e.g., MAPK pT202 Y204, MEK1 pS217 S221), mammalian target of rapamycin (mTOR) pathway proteins (e.g., mTOR pS2448, PKCPANβII pS660, GSK3αβ pS21S9, FOXM1), anti-apoptotic proteins (e.g., BAD pS112, PDCD4), and DNA damage response (e.g., MSH2, CDK1 pY15, and CHK1 pS296) (Supplementary Table S5). Interestingly, other anti-apoptotic genes such as BIM, BAK, or BCL2A1 are significantly lower in Y163C-positive samples. In comparison between R282 hotspot mutations and group 1 mutations, mTOR was significantly higher in samples with R282 hotspot mutations, while MYOSINIIA was higher in samples with R175, C176, S241, and G245 hotspot mutations (Supplementary Table 5). Transglutaminase, p38MAPK, or extracellular signal-regulated kinase (ERK) were higher in samples with R175, C176, C238, S241, G245, and R273 hotspot mutations. When we compared samples with G266 mutations to samples with group 1 mutations, we found that the expression of cMETpY1235, NOTCH1, and/or YAPpS127 is higher in G266 mutation-positive samples.

## Discussion

Our analysis yields new insights about the impact of different *TP53* mutations on survival time in ovarian cancer. We found no association among the type of mutations, which was consistent with a previous report.^14^ There are multiple potential functional outcomes of *TP53* mutations: LoF, gain-of-function, dominant negative, and no effect. Missense mutations have the potential to fall into each of the classes.^31–33^ Thus comparisons between different groups of *TP53* missense mutations have the potential to identify different associations with outcomes. We also have compared missense mutations with truncated as well as in-frame mutations. We did not find significant difference in survival between the truncated mutations and different classes of mutations. This is in part due to assessing all missense mutations as a single group. Truncating mutations, including nonsense, frameshift and splice site mutations, are usually predicted to cause LoF.^34^ Nonsense mutations lead to early termination codons, which are frequently targets of nonsense-mediated decay (NMD). However, not all nonsense mutations result in NMD. Not all truncating mutations lead to LoF. For example, a rare nonsense mutation of *TP53* (378C>G) creates a stop codon, which escapes NMD and premature termination of translation does not occur due to an alternative 3’ splice site. The subsequent p53 protein product is able to induce apoptosis by activating p21.^35^ Some truncation mutations can result in stable proteins that mediate some if not all of the effects of p53. Thus truncation mutations may not necessarily result in LoF of *TP53*. Our study provides strong evidence that *TP53* mutations are not functionally equivalent in terms of outcome in HGSOC and support the recent report that missense mutations of *TP53* (R248W and R175H) drive tumorigenesis and metastasis differently in mouse models.^36^

In fact, we identified two groups of hotspot mutations with different effects on outcomes. Group 1 consisted of 15 hotspot mutations; R175, C176, H179, H193, I195, Y220, Y234, C238, S241, G245, R248, R273, C275, P278, and D281, while group 2 contained three hotspot mutations; Y163C, G266, and R282. Group 1 mutations were associated with better OS and RFS than the group 2 hotspot mutations. Importantly, Group 2 hotspot mutations were a strong predictor for worsened OS in the training, validation, and combined sets as well as for worsened RFS in the training and combined data sets. When we tested each hotspot mutation individually, we found that hotspot mutations also had different impact on survival time. For instance, samples with R273 mutations have prolonged OS compared to those with Y163C, G266, and R282 mutations. Similarly, patients with R175, C176, Y220, S241, G245, D281, and C238 mutations showed better OS than the Y163C-, G266-, and R282-mutated cases. Furthermore, R273-mutated cases were associated with better OS time than the patients with R248 mutations. This later finding is consistent with an earlier report.^37^ However, no significant association was found between patients with R273 and cases with R175, C176, I195, Y220, C238, S241, G245, C275, and P278 mutations on survival time (OS and RFS). This data indicates the importance to evaluate the impact of each mutation on functional events, therapeutic sensitivity, and patient outcomes separately.

Missense mutations most often occur in the core DBD and rarely in non-DNA-binding domains. Residues in DBD play important roles in (1) DNA contact with mutations in these residues resulting in loss of DNA binding or in (2) stable folding of the core domain with mutations in these residues impairing correct folding of the core domain. Therefore, missense mutations in the DBD further can be classified into two groups: contact and structural mutations.^10^ R273, R248, and R282 are grouped as contact mutations. Y163, R175, C176, H179, C238, C242, and G245 are grouped as structural mutations.^10,38^ I195, Y220, and Y234 mutations are important for the structure of the short loops.^10,38,39^ Interestingly, in our study R273 was associated with longer OS than the other two contact mutations R248 and R282. Similarly, Y163C mutations were associated with a shorter OS than the other structural hotspot mutations. Collectively, these data indicate that each mutation has distinct impact on survival regardless of the type or structural classification of mutations or location of mutation.

Similar to ovarian cancer, in breast cancer different hotspot mutations have been associated with survival. For example, mutations at H179 and R248W were reported to be associated with reduced survival, while G245S and Y220C mutations were associated with better survival compared with any other missense mutations.^40^ No significant impact on survival was found by grouping samples based on structure, function, or conservation feature of mutant protein in breast cancer.^40^

When we tested for differently expressed proteins between the samples with hotspot mutations, six proteins were overexpressed in R273 mutant samples compared with samples with R248 mutations: ASNS, N-cadherin, PCNA, RAD51, BCL2A1, and PYGL. Asparagine synthetase (ASNS) catalyses asparagine synthesis from aspartate and glutamine.^41^ ASNS has been shown to be transcriptionally activated by R273H^42^; while PCNA was transcriptionally activated by both R273H and R248W mutations.^8^ Thus our results support the earlier report suggesting that R273H transcriptionally activates ASNS and induces proliferation.^8,42^ Low expression of ASNS is a poor prognostic (shorter OS) factor in hepatocellular cancer^43^ and in rectal cancer.^44^

When we compared samples with Y163C mutations to samples with hotspot mutations in group 1, we found that cell signalling and proliferation, proteins itself, and phosphorylation are higher in samples with Y163C: Cyclin B1, GSK3abpS21S9, MAPKpT202Y204, MEK1pS217S221, mTORpS2448, BADpS112, PKCpanbetaIpS660, AKTpT308, and CDK1pY15. MTOR higher in R282 and cMETpY1235 and NOTCH1 are higher in samples with G266, suggesting that mutational events in this poor prognosis group may lead to activation of the AKT or MAPK, mTOR, or NOTCH signalling pathways. This supports earlier observations that activation of the Akt/mTOR pathway contributes to cisplatin resistance in ovarian cancer cells.^45^ Resistance to cisplatin, the key drug in treatment of HGSOC, may explain the worsened outcomes associated with Y163C.

Mutant p53 has diverse impact of tumorigenesis: DNA synthesis and proliferation; survival; chemoresistance; gene amplification; abnormal centrosomes and spindle checkpoints; somatic cell reprogramming; angiogenesis, migration, and invasion; and metabolic remodelling.^46^ Most of the mutations that deactivate p53 block the ability of the protein to bind to its targets and hence interfere with transcriptional activation of these genes. However, a number of *TP53* mutations alter the function and binding partners of p53.^8^ Our data are consistent with earlier reports that *TP53* mutations can regulate different proteins or signalling pathways. Therefore, it is not surprising that hotspot mutations can have different implications on outcome of disease and in response to chemotherapy. In conclusion, our data support the behaviour of each *TP53* mutation being different, requiring evaluation of each mutation separately for associations with survival and response to therapy.

## Supplementary information

Supplementary Table and Figure Legends, and Tables and Figures

## Data Availability

TCGA generated data set that was analysed during the current study is available from the following URLS: http://portal.gdc.cancer.gov, http://Xena.ucsc.edu, https://www.tcpaportal.org/tcpa, and validation data set is available from the previous publications (Supplementary Table S1) and corresponding author on reasonable request.
