# Supplementary material for: Clinical relevance of TP53 hotspot mutations in high-grade serous ovarian cancers
Source: Br J Cancer. 2019 Nov 29;122(3):405–12. doi: 10.1038/s41416-019-0654-8 (PMC7000721; doi:10.1038/s41416-019-0654-8)
Supplement: Supplementary file 1 — Supplementary Table and Figure Legends, and Tables and Figures [file 41416_2019_654_MOESM1_ESM.pdf]

Supplementary Table Legends:

Supplementary Table S1. Detailed information about samples used in this study.

Supplementary Table S2. Patient demographics are used in survival analysis.

Supplementary Table S3. Univariate OS and RFS analyses of type of *TP53* mutations in training, validation and combined sets of HGSOC.

Supplementary Table S4. Univariate OS and RFS analyses of *TP53* hotspot mutations in combined set of HGSOC.

Supplementary Table S5. Differentially expressed proteins between hotspot mutations.

Supplementary Figure legend:

Supplementary Figure S1. Kaplan-Meier plot of overall survival probability for patients with all *TP53* hotspot mutations.

Supplementary Table S1. High-grade serous ovarian cancer samples used in this study

TP53 mutation and clinical data	Sample size	Ref
TCGA	468	[1], http://Xena.ucsc.edu
Ahmed et al	118	[2]
Patch et al	12	[3-5]
Emanuel et al Garsed et al	12	[4, 6-8]
Hayano et al	13	[9]
Chien et al	15	[10]
Beltrame et al	7	[11]
Schwarz et al	15	[12]
Wojnarowich et al	81	[13]
Jean et al	52	[14]

References:

1. The Cancer Genome Atlas Network. Integrated genomic analyses of ovarian carcinoma. Nature 2011, 474, 609-15.
2. Ahmed AA, Etemadmoghadam D, Temple J, et al. Driver mutations in TP53 are ubiquitous in high grade serous carcinoma of the ovary. J Pathol 2010, 221, 49-56.
3. Patch AM, Christie EL, Etemadmoghadam D, et al. Whole-genome characterization of chemoresistant ovarian cancer. Nature 2015, 521, 489-94.
4. Tothill RW, Tinker AV, George J, et al. Novel molecular subtypes of serous and endometrioid ovarian cancer linked to clinical outcome. Clin Cancer Res 2008, 14, 5198-208.
5. Wang ZC, Birkbak NJ, Culhane AC, et al. Profiles of genomic instability in high-grade serous ovarian cancer predict treatment outcome. Clin Cancer Res 2012, 18, 5806-15.
6. Garsed DW, Alsop K, Fereday S, et al. Homologous Recombination DNA Repair Pathway Disruption and Retinoblastoma Protein Loss Are Associated with Exceptional Survival in High-Grade Serous Ovarian Cancer. Clin Cancer Res 2018, 24, 569-580.
7. Baumbusch LO, Helland A, Wang Y, et al. High levels of genomic aberrations in serous ovarian cancers are associated with better survival. PLoS One 2013, 8, e54356.
8. Emmanuel C, Chiew YE, George J, et al. Genomic classification of serous ovarian cancer with adjacent borderline differentiates RAS pathway and TP53-mutant tumors and identifies NRAS as an oncogenic driver. Clin Cancer Res 2014, 20, 6618-30.
9. Hayano T, Yokota Y, Hosomichi K, et al. Molecular characterization of an intact p53 pathway subtype in high-grade serous ovarian cancer. PLoS One 2014, 9, e114491.
10. Chien J, Sicotte H, Fan JB, et al. TP53 mutations, tetraploidy and homologous recombination repair defects in early stage high-grade serous ovarian cancer. Nucleic Acids Res 2015, 43, 6945-58.
11. Beltrame L, Di Marino M, Fruscio R, et al. Profiling cancer gene mutations in longitudinal epithelial ovarian cancer biopsies by targeted next-generation sequencing: a retrospective study. Ann Oncol 2015, 26, 1363-71.
12. Schwarz RF, Ng CK, Cooke SL, et al. Spatial and temporal heterogeneity in high-grade serous ovarian cancer: a phylogenetic analysis. PLoS Med 2015, 12, e1001789.
13. Wojnarowicz PM, Oros KK, Quinn MC, et al. The genomic landscape of TP53 and p53 annotated high grade ovarian serous carcinomas from a defined founder population associated with patient outcome. PLoS One 2012, 7, e45484.
14. JeanS, Li j, Katsaros D, Wubbenhorst B, Maxwell KN, Fishbein L, et al. Paclitaxel is necessary for improved survival in epithelial ovarian cancers with homologous recombination gene mutations. Oncotarget 2016, 7, 48577-48585.

Supplementary Table S2. Patient demographics are used in survival analysis.

Data Sets		Age							Stage					
	**Mutation**	**Total**	**≤55**	**>55**	**NA**	***p***	***q***	***p***	**I&II**	**III&IV**	**NA**	***p***	***q***	***p***
**TCGA**		**466**	**186**	**279**	1				**37**	**425**	4			
	OS (median, months)	32.67	36.73	29.53	6	0.009	0.018		30.32	33.33	6	0.995	0.995	
	RFS (median, months)	14.32	16.93	13.13	6	0.031	0.031		19	14.2	6	0.040	0.080	
	Missense	60.52%	61.29%	60.22%	0	0.816	0.952		51.35%	61.88%	0	0.208	0.728	
	Nonsense	10.73%	12.37%	9.32%	1	0.295	0.782		18.92%	9.88%	1	0.087	0.420	
	Frameshift	15.24%	16.13%	14.70%	0	0.674	0.944		13.51%	14.82%	3	0.829	0.965	
	Splice site	10.52%	7.53%	12.54%	0	0.084	0.782		10.81%	10.59%	0	0.966	0.966	
	In frame	3.22%	2.69%	3.58%	0	0.593	0.922	0.416	5.41%	3.06%	0	0.440	0.965	0.402
	Hotspot mutations	27.04%	25.27%	28.32%	0	0.290	0.782	0.637	35.14%	26.82%	0	0.322	0.728	0.563
**Validation**		**325**	**97**	**215**	13				**41**	**284**				
	OS (median, months)	34	34	33		0.533	0.669		45	31		< 0.0001	<0.0001	
	RFS (median, months)	18	18	18		0.669	0.669		39	15		< 0.0001	<0.0001	
	Missense	59.69%	57.73%	60.93%	7	0.594	0.717		63.41%	59.15%	0	0.604	0.796	
	Nonsense	12.00%	9.28%	13.02%	2	0.344	0.541		9.76%	12.32%	0	0.637	0.796	
	Frameshift	20.00%	27.84%	17.21%	1	**0.031**	0.155		17.07%	20.42%	0	0.616	0.796	
	Splice site	7.08%	5.15%	7.91%	1	0.379	0.541		7.32%	7.04%	0	0.949	0.949	
	In frame	1.23%	0.00%	0.93%	2	0.341	0.541	0.184	2.44%	1.06%	0	0.453	0.796	0.899
	Hotspot mutations	20.62%	16.49%	22.33%	3	0.560	0.717	0.071	24.39%	20.07%	0	0.807	0.949	0.454
**Combined**		**791**	**283**	**494**	14				**78**	**709**	4			
	OS (median, months)	33	35.1	31		0.014	0.028		41	32.57		0.012	0.012	
	RFS (median, months)	15.33	16.97	14.65		0.193	0.193		28.93	14.7		< 0.0001	<0.0001	
	Missense	60.05%	60.07%	60.32%	7	0.941	0.942		56.41%	60.79%	0	0.485	0.965	
	Nonsense	11.38%	11.31%	11.13%	3	0.941	0.942		14.10%	11.00%	1	0.362	0.965	
	Frameshift	17.19%	20.14%	15.79%	1	0.124	0.595		15.38%	17.07%	3	0.707	0.965	
	Splice site	8.98%	6.71%	10.32%	1	0.091	0.594		8.97%	9.03%	0	0.955	0.965	
	In frame	2.40%	1.77%	2.43%	2	0.544	0.888	0.283	3.85%	2.26%	0	0.361	0.965	0.770
	Hotspot mutations	38.69%	36.75%	39.47%	7	0.463	0.888	0.407	38.46%	38.79%	1	0.452	0.965	0.991

Supplementary Table S3. Univariate OS and RFS analyses of type of *TP53* mutations in training, validation and combined sets of HGSOC.

	Training set (TCGA)				Validation set				Combined data			
**Covariates**	**HR**	***p***	***q***	**95%CI**	**HR**	***p***	***q***	**95%CI**	**HR**	***p***	***q***	**95%CI**
**OS**												
Missense vs nonsense	0.99	0.974	0.974	0.66-1.48	0.67	0.103	0.275	0.42-1.08	0.85	0.291	0.881	0.62-1.15
Missense vs frameshift	0.97	0.718	0.947	0.81-1.16	0.92	0.366	0.418	0.78-1.10	0.96	0.492	0.904	0.84-1.08
Missense vs splice site	1.05	0.426	0.936	0.93-1.19	1.14	0.158	0.316	0.95-1.36	1.06	0.236	0.881	0.96-1.17
Nonsense vs splice site	1.08	0.559	0.947	0.84-1.38	1.49	**0.023**	0.184	1.06-2.11	1.19	0.088	0.780	0.97-1.45
Nonsense vs frameshift	0.94	0.813	0.947	0.57-1.54	1.24	0.444	0.444	0.72-2.13	1.08	0.690	0.904	0.75-1.55
Splice site vs frameshift	1.24	0.364	0.936	0.78-1.98	1.83	0.052	0.208	0.99-3.36	1.33	0.120	0.780	0.93-1.89
Splice site vs inframe	1.15	0.705	0.947	0.56-2.32					1.20	0.563	0.904	0.64-2.27
Nonsense vs inframe	1.10	0.432	0.936	0.87-1.40					0.88	0.695	0.904	0.47-1.66
Missense vs inframe	1.07	0.388	0.936	0.91-1.26					1.01	0.981	0.981	0.57-1.79
Frameshift vs inframe	1.17	0.371	0.936	0.83-1.67					0.91	0.769	0.909	0.50-1.68
Missense vs truncated	1.05	0.778	0.947	0.76-1.44	1.15	0.322	0.418	0.87-1.53	1.04	0.655	0.904	0.86-1.26
Missense (without hotspots) vs truncated	0.98	0.874	0.947	0.76-1.26	1.27	0.203	0.325	0.88-1.84	1.23	0.339	0.881	0.88-1.43
Inframe vs truncated	1.05	0.432	0.936	0.92-1.20					1.03	0.909	0.981	0.58-1.86
**RFS**												
Missense vs nonsense	0.97	0.903	0.908	0.66-1.44	0.89	0.577	0.820	0.58-1.35	0.92	0.580	0.932	0.69-1.23
Missense vs frameshift	1.01	0.908	0.908	0.85-1.20	1.05	0.526	0.820	0.89-1.24	1.03	0.645	0.932	0.91-1.16
Missense vs splice site	0.95	0.479	0.890	0.84-1.09	1.04	0.696	0.820	0.86-1.25	0.98	0.733	0.953	0.88-1.09
Nonsense vs splice site	0.95	0.717	0.908	0.74-1.23	1.13	0.455	0.820	0.82-1.57	1.01	0.884	0.990	0.83-1.24
Nonsense vs frameshift	1.07	0.774	0.908	0.67-1.73	1.29	0.289	0.820	0.80-2.09	1.17	0.353	0.830	0.84-1.64
Splice site vs frameshift	0.83	0.456	0.890	0.51-1.35	1.08	0.811	0.820	0.59-1.96	0.9	0.570	0.932	0.63-1.30
Splice site vs inframe	0.73	0.463	0.890	0.32-1.68					1.38	0.356	0.830	0.69-2.76
Nonsense vs inframe	0.88	0.363	0.890	0.67-1.16					1.35	0.383	0.830	0.69-2.65
Missense vs inframe	0.90	0.253	0.890	0.74-1.08					1.47	0.232	0.830	0.78-2.76
Frameshift vs inframe	0.80	0.268	0.890	0.53-1.19					1.55	0.190	0.830	0.81-2.98
Missense (all) vs truncated	1.04	0.750	0.908	0.81-1.33	0.97	0.820	0.820	0.74-1.27	1.00	0.990	0.990	0.83-1.20
Missense (without hotspots) vs truncated	0.97	0.853	0.908	0.71-1.33	1.09	0.634	0.820	0.77-1.54	1.00	0.968	0.990	0.80-1.27
Inframe vs truncated	0.93	0.322	0.890	0.79-1.08					0.69	0.260	0.830	0.37-1.31

Supplementary Table S4. Univariate OS and RFS analysis of *TP53* hotspot mutations in combined set of HGSOC.

	OS				RFS			
**Covariates**	**HR**	***p***	***q***	**95%CI**	**HR**	***p***	***q***	**95%CI**
R273C vs R273L	0.61	0.446	0.446	0.17-2.16	0.22	**0.029**	0.158	0.05-0.86
R273 vs Y163C	1.23	**<0.0001**	**<0.0001**	1.10-1.38	1.11	0.102	0.213	0.98-1.25
R273 vs G266	1.14	**<0.0001**	**<0.0001**	1.06-1.22	1.07	0.119	0.219	0.98-1.15
R273 vs R282	1.08	**0.007**	**0.023**	1.02-1.15	1.06	**0.016**	0.158	1.01-1.12
R175 vs Y163C	1.35	**0.001**	**0.012**	1.14-1.60	1.15	0.117	0.219	0.97-1.36
R175 vs G266	1.93	**<0.0001**	**<0.0001**	1.08-1.31	1.09	0.116	0.219	0.98-1.20
R175 vs R282	1.12	**0.004**	**0.018**	1.04-1.20	1.07	**0.025**	0.158	1.01-.114
C176 vs Y163C	0.50	**0.002**	**0.017**	0.32-0.77	1.58	**0.027**	0.158	1.05-2.38
C176 vs G266	4.64	**0.006**	**0.021**	1.55-13.86	1.20	**0.042**	0.158	1.01-1.42
C176 vs R282	1.32	**0.011**	**0.028**	1.07-1.64	1.14	**0.010**	0.158	1.03-1.25
S241 vs Y163C	0.73	**0.006**	**0.021**	0.58-0.91	0.89	0.143	0.228	0.75-1.04
S241 vs G266	0.51	**0.004**	**0.018**	0.32-0.81	0.75	0.144	0.228	0.52-1.10
S241 vs R282	2.80	**0.014**	**0.032**	1.23-6.34	2.01	**0.018**	0.158	1.13-3.61
R248 vs Y163C	1.15	**0.033**	**0.046**	1.01-1.31	1.09	0.220	0.314	0.95-1.26
R248 vs G266	1.11	**0.011**	**0.028**	10.2-1.20	1.05	0.266	0.360	0.96-1.15
R248 vs R282	1.06	0.085	0.089	0.99-1.12	1.06	0.059	0.158	0.99-1.12
Y220 vs Y163C	1.28	**0.014**	**0.032**	1.05-1.56	1.16	0.173	0.257	0.94-1.42
Y220 vs G266	1.17	**0.003**	**0.017**	1.05-1.30	1.07	0.225	0.314	0.96-1.20
Y220 vs R282	1.08	**0.045**	0.058	1.00-1.17	1.08	**0.035**	0.158	1.01-1.15
I195 vs Y163C	1.34	**0.042**	0.057	1.01-1.77	1.14	0.360	0.449	0.86-1.50
I195 vs G266	1.14	0.051	0.062	0.99-1.29	1.06	0.371	0.449	0.93-1.22
I195 vs R282	1.07	0.137	0.140	0.98-1.18	1.06	0.164	0.251	0.98-1.14
G245 vs Y163C	2.61	**0.003**	**0.017**	1.39-4.91	1.77	**0.044**	0.158	1.02-3.09
G245 vs G266	1.32	**0.003**	**0.017**	1.10-1.58	1.19	0.062	0.158	0.99-1.43
G245 vs R282	1.19	**0.010**	**0.028**	1.04-1.35	1.17	**0.003**	0.138	1.06-1.31
H179 vs Y163C	0.22	**0.022**	**0.037**	0.06-0.80	0.56	0.370	0.449	0.16-1.99
H179 vs G266	1.67	**0.018**	**0.035**	1.09-2.56	1.26	0.291	0.382	0.82-1.93
H179 vs R282	1.17	0.065	0.073	0.99-1.38	1.13	0.124	0.219	0.97-1.32
P278 vs Y163C	0.52	**0.043**	0.057	0.28-0.98	0.78	0.409	0.482	0.42-1.42
P278 vs G266	1.71	0.066	0.073	0.97-3.02	1.15	0.666	0.747	0.61-2.19
P278 vs R282	1.19	0.085	0.089	0.98-1.46	1.19	0.058	0.158	0.99-1.42
H193 vs Y163C	0.74	**0.033**	**0.046**	0.56-0.98	0.98	0.858	0.881	0.77-1.24
H193 vs G266	0.18	**0.015**	**0.033**	0.05-0.72	0.95	0.936	0.936	0.29-3.14
H193 vs R282	1.43	0.054	0.064	0.99-2.05	1.03	0.829	0.881	0.78-1.35
C275 vs Y163C	0.78	**0.033**	**0.046**	0.62-0.98	0.84	0.100	0.213	0.67-1.04
C275 vs G266	0.48	**0.021**	**0.037**	0.26-0.90	0.61	0.132	0.225	0.32-1.16
C275 vs R282	1.56	0.067	0.073	0.97-2.50	1.43	0.072	0.166	0.97-2.11
D281 vs Y163C	0.42	**0.017**	**0.034**	0.21-0.86	0.57	0.051	0.158	0.33-1.00
D281 vs G266	11.38	**0.023**	**0.038**	1.40-92.63	4.85	0.061	0.158	0.93-25.27
D281 vs R282	1.48	**0.033**	**0.046**	1.03-2.13	1.33	**0.034**	0.158	1.02-1.73
C238 vs Y163C	0.75	**0.005**	**0.021**	0.61-0.92	0.97	0.744	0.815	0.83-1.14
C238 vs G266	0.58	**0.010**	**0.028**	0.38-0.88	0.97	0.862	0.881	0.66-1.41
C238 vs R282	2.20	**0.028**	**0.044**	1.09-4.44	1.19	0.521	0.599	0.70-2.01
Y234 vs Y163C	0.89	**0.011**	**0.028**	0.81-0.97	0.92	0.069	0.166	0.85-1.01
Y234 vs G266	0.90	**0.010**	**0.028**	0.84-0.98	0.92	0.058	0.158	0.84-1.00
Y234 vs R282	5.15	0.051	0.062	0.99-26.73	4.32	**0.036**	0.158	1.10-16.97

Supplementary Figure S1

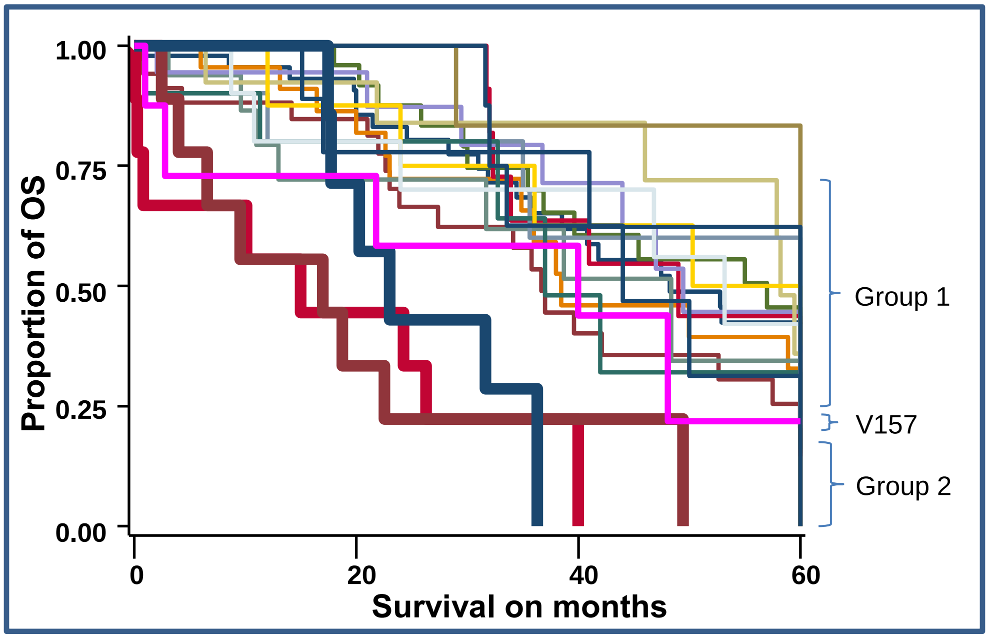

Supplementary Table S5-1. Differentially expressed proteins between R273 and R248 hotspot mutations

Proteins	Median R273	Median R248	*p*	*q*
ASNS	0.50642	0.046715	0.033947	0.039286
CJUNPS73	0.075365	0.15423	0.039286	0.039286
NCADHERIN	0.54404	0.11639	0.026599	0.039286
PCNA	0.0369	-0.19117	0.022621	0.039286
RAD51	-0.02955	-0.12785	0.027404	0.039286
MYOSINIIAPS1943	0.096777	0.25636	0.029551	0.039286
NDRG1PT346	-0.20926	0.41861	0.029351	0.039286
BCL2A1	0.18539	-0.0434	0.03704	0.039286
PYGL	0.50642	0.046715	0.033947	0.039286
NRF2	0.075365	0.15423	0.039286	0.039286

Supplementary Table S5-2. Differentially expressed proteins between R273 and R282 hotspot mutations

Proteins	Median R273	Median R282	*p*	*q*
MTOR	0.25709	0.38439	0.003918	0.011754
PEA15	0.131798	-0.39006	0.000501	0.003006
NRAS	-0.03993	0.043484	0.024776	0.024776
TRANSGLUTAMINASE	-0.35085	-0.61802	0.015056	0.020089
MYOSINIIA	0.036432	-0.24965	0.016741	0.020089
ERK2	0.080393	-0.63057	0.009089	0.018178

Supplementary Table S5-3. Differentially expressed proteins between R273 and Y163C hotspot mutations

Proteins	Median R273	Median Y163C	*p*	*q*
BAK	-0.2383	-0.4763	0.006147	0.016685
BIM	0.350825	-0.26626	0.00044	0.005573
CMETPY1235	0.113150	-0.02097	0.034191	0.043309
CYCLINB1	0.316945	0.94647	0.009817	0.021944
EGFR	-0.20423	-0.41274	0.000366	0.005573
EGFRPY1173	-0.10766	-0.17253	0.001584	0.015048
ERK2	0.080399	-0.36597	0.046477	0.047733
FOXO3A	0.032892	-0.15627	0.043963	0.047147
GSKALPHABETAPS21S9	-0.42551	0.040349	0.03051	0.041537
MAPKPT202Y204	0.347425	1.2262	0.003622	0.015982
MEK1PS217S221	-0.17331	0.51562	0.021043	0.038012
MTORPS2448	0.18221	0.40045	0.004618	0.015982
P27	-0.11146	-0.37691	0.023125	0.038012
P90RSKPT359S363	0.165305	0.482335	0.024959	0.038012
PEA15	0.131798	-0.23834	0.004923	0.015982

PR	-0.09387	-0.24385	0.004051	0.015982
STATPY705	0.11217	0.478415	0.018166	0.034515
STATHMIN	-0.00672	-0.06878	0.022299	0.038012
BADPS112	0.28137	0.62223	0.00335	0.015982
FASN	-0.47288	-1.35115	0.005047	0.015982
FOXM1	0.250255	1.061355	0.005882	0.016685
GSK3PS9	-0.27432	0.154786	0.035467	0.043476
P27PT198	0.023229	-0.08301	0.029956	0.041537
PDCD4	0.50407	1.20225	0.002434	0.015982
PKCPANBETAIIPS660	-0.064	0.42061	0.025008	0.038012
RAB11	-0.03824	-0.16609	0.01326	0.02652
ERCC1	-0.05501	-0.15622	0.030606	0.041537
MSH2	0.223	0.577635	0.037953	0.043703
AXL	-0.15884	0.131862	0.037363	0.043703
CDK1PY15	-0.18633	0.193455	0.003837	0.015982
CD26	-0.09182	-0.18406	0.048874	0.048874
CHK1PS296	0.030596	0.140965	0.008159	0.019378
ERCC5	-0.27656	0.206275	0.011024	0.023272
P16INK4A	1.2558	1.65385	0.044666	0.047147
CTLA4	-0.2383	-0.4763	0.006615	0.016757
MACC1	0.350825	-0.26626	0.00044	0.005573
NRF2	0.075365	0.34139	0.041502	0.046385
THYMIDILATESYNTHASE	0.011315	-0.02097	0.034191	0.043309

Supplementary Table S5-4. Differentially expressed proteins between R273 and G266 hotspot mutations

	Median			
Proteins	R273	G266	*p*	*q*
CMETPY1235	0.011315	0.10229	0.033221	0.039076
NOTCH1	-0.26107	0.132825	0.031435	0.039076
YAP	0.241725	-0.30376	0.020909	0.039076
ANNEXINVII	-0.04412	-0.24813	0.03225	0.039076
ANNEXIN1	0.36209	-0.45296	0.0273	0.039076
JAB1	0.05345	-0.16196	0.019834	0.039076
AXL	-0.15884	0.19755	0.049351	0.039076
THYMIDILATESYNTHASE	0.011315	0.10229	0.033221	0.039076
EPPK1	2.17695	0.058108	0.034734	0.039076

Supplementary Table S5-5. Differentially expressed proteins between R175 and R248 hotspot mutations

	Median			
Proteins	R175	R248	*p*	*q*
CIAP	0.092068	-0.01701	0.039247	0.043608
ER-ALPHA	1.0759	1.68795	0.049641	0.049641
INPP4B	-0.18453	0.126813	0.009644	0.025156

P53	0.407345	0.702545	0.013718	0.025156
VEGFR2	0.20894	0.006479	0.016744	0.025156
PDK1	0.11001	-0.00669	0.017955	0.025156
SF2	0.007797	-0.09893	0.016261	0.025156
TUBERINpT1462	0.14657	-0.25397	0.014996	0.025156
MSH2	-0.07729	0.257855	0.020125	0.025156
MSH6	0.032965	0.37794	0.0123	0.025156

Supplementary Table S5-6. Differentially expressed proteins between R175 and R282 hotspot mutations

	Median			
Proteins	R175	R282	*p*	*q*
HER3	0.15521	-0.0185	0.019599	0.025766
KU80	-0.27715	0.15987	0.011964	0.019142
MTOR	0.039794	0.38439	2.27E-06	6.32E-05
NOTCH1	-0.27005	-0.07012	0.000418	0.002229
P38MAPK	0.006595	-0.34	0.027446	0.030013
PAI	0.059292	-0.5291	0.016099	0.023417
PEA15	0.063432	-0.39006	0.000203	0.001624
YAPPS127	0.54093	0.23633	0.003293	0.011085
CD20	-0.09122	-0.19592	0.007127	0.01629
FOXM1	0.07953	0.4515	0.003464	0.011085
RICTORPT1135	0.11081	-0.01293	0.028137	0.030013
TRANSGLUTAMINASE	-0.46113	-0.61802	0.020935	0.025766
AXL	0.12286	-0.11121	0.00696	0.01629
MYOSINIIA	0.055057	-0.24695	0.010374	0.018443
DUSP4	-0.32201	-0.1447	0.009267	0.018447
PDK1	0.069959	-0.21678	0.048367	0.048367

Supplementary Table S5-7. Differentially expressed proteins between R175 and Y163C hotspot mutations

	Median			
Proteins	R175	Y163C	*p*	*q*
AKTPT308	-0.74848	-0.42893	0.007022	0.013068
AR	0.52445	-0.12727	0.045501	0.048277
BAK	-0.20063	-0.4763	0.007319	0.013068
BIM	0.3781	-0.26626	0.004997	0.012685
CYCLINB1	-0.04788	0.94647	0.000548	0.006191
EGFR	-0.11537	-0.41274	0.000807	0.006191
EGFRPY1173	-0.05538	-0.17253	0.002894	0.00955
FOXO3A	0.037577	-0.15627	0.011733	0.016834
GSK3ALPHABETAPS21S9	-0.57847	0.040349	0.025229	0.030835
KU80	-0.27715	0.085044	0.008215	0.013288
MAPKPT202Y204	0.7065	1.2262	0.008456	0.013288

MEK1PS217S221	-0.03664	0.51562	0.049406	0.049406
MTORPS2448	0.2559	0.40045	0.007501	0.013068
P27	0.031651	-0.37691	0.00085	0.006191
PEA15	0.063432	-0.23836	0.002004	0.008267
PR	-0.13142	-0.24385	0.012288	0.016896
SYK	0.77143	1.2806	0.0155	0.02046
BADPS112	0.36245	0.62223	0.00436	0.012685
CD20	-0.09122	-0.18073	0.016711	0.02121
FASN	-0.53817	-1.35115	0.007524	0.013068
FOXM1	0.07953	1.061355	0.000938	0.006191
P27PT198	0.035527	-0.08301	0.001141	0.006273
PDCD4	0.58143	1.20225	0.008993	0.013489
PKCPANBETAIIPS660	0.042578	0.42061	0.046814	0.048277
MSH2	-0.0839	0.577635	0.000922	0.006191
MSH6	-0.00309	0.78435	0.036217	0.041212
CDK1PY15	-0.18491	0.193455	0.002468	0.009049
CD26	-0.07492	-0.18406	0.034208	0.040317
CHK1PS296	0.016797	0.140965	0.001873	0.008267
OXPHOSCOMPLX V SUBUNIT B	-0.74848	-0.42893	0.007022	0.013068
PYGBAB2	0.52445	-0.12727	0.045501	0.048277
CTLA4	-0.20063	-0.4763	0.007319	0.013068
MACC1	0.3781	-0.26626	0.004997	0.012685

Supplementary Table S5-8. Differentially expressed proteins between R175 and G266 hotspot mutations

	Median			
Proteins	R175	G266	*p*	*q*
BID	-0.12567	0.2221	0.048155	0.049718
CMETPY1235	0.014106	0.10229	0.029754	0.049718
NOTCH1	-0.27005	0.132825	0.006298	0.049718
YAP	0.1692	-0.30376	0.027761	0.049718
YAPPS127	0.54093	-0.11524	0.038994	0.049718
ANNEXINVII	-0.05076	-0.24813	0.04329	0.049718
HEREGULIN	0.019876	-0.05316	0.049718	0.049718
JAB1	0.077014	-0.16196	0.013983	0.049718
LCN2A	-0.12567	0.2221	0.048155	0.049718

Supplementary Table S5-9. Differentially expressed proteins between C176 and R282 hotspot mutations

	Median			
Proteins	C176	R282	*p*	*q*
CAVEOLIN1	-0.27521	-1.6251	0.026711	0.036276
MTOR	0.056779	0.38439	0.002925	0.016088

MTORPS2448	-0.08231	0.25577	0.039901	0.039901
P38MAPK	-0.02582	-0.34	0.022858	0.036276
PEA15	0.19821	-0.39006	0.001562	0.016088
YAPPS127	0.72934	0.23633	0.02968	0.036276
FOXO3APS318S321	0.09696	-0.12485	0.027647	0.036276
TRANSGLUTAMINASE	-0.36487	-0.61802	0.006309	0.023133
JAB1	-0.06948	0.07454	0.019815	0.036276
MYOSINIIA	-0.01697	-0.24695	0.037839	0.039901
DUSP4	-0.43984	-0.1447	0.011732	0.032263

Supplementary Table S5-10. Differentially expressed proteins between C176 and Y163 hotspot mutations

	Median			
Proteins	C176	Y163	*p*	*q*
BAK	-0.30285	-0.4763	0.016975	0.035025
BIM	0.70943	-0.26626	0.001141	0.009508
CYCLINB1	0.26474	0.94647	0.027053	0.035388
EGFR	-0.25223	-0.41274	0.017923	0.035025
EGFRPY1173	-0.05287	-0.17253	0.002591	0.011575
HSP70	0.2186	-0.35642	0.018213	0.035025
IRS1	-0.09782	-0.34309	0.02831	0.035388
JNK2	-0.17025	-0.64792	0.022708	0.035388
MTORPS2448	-0.08231	0.40045	0.000146	0.00365
NOTCH1	-0.15221	-0.40967	0.023773	0.035388
P27	0.18617	-0.37691	0.008427	0.026334
P90RSKPT359S363	0.12768	0.482535	0.027911	0.035388
PEA15	0.19821	-0.23836	0.010573	0.029369
STAT3PY705	0.25743	0.478415	0.027905	0.035388
BADPS112	0.22714	0.62223	0.002268	0.011575
FASN	-0.72136	-1.35115	0.02049	0.035388
FOXM1	0.62208	1.061355	0.040697	0.044144
P27PT198	0.16132	-0.08301	0.002778	0.011575
PDCD4	0.50861	1.20225	0.005923	0.021154
PKCPANBETAIIPS660	-0.03067	0.42061	0.046096	0.046096
CDK1PY15	-0.16403	0.193455	0.042378	0.044144
CHK1PS296	0.011275	0.140965	0.033789	0.040225
P16INK4A	1.2083	1.65385	0.036638	0.041634
CTLA4	-0.30285	-0.4763	0.016975	0.035025
MACC1	0.70943	-0.26626	0.001141	0.009508

Supplementary Table S5-11. Differentially expressed proteins between C176 and G266 hotspot mutations

	Median			
Proteins	C176	G266	*p*	*q*
MTORPS2448	-0.08231	0.233	0.042533	0.047231
YAP	0.25017	-0.30376	0.015602	0.046806
YAPPS127	0.72934	-0.11524	0.047231	0.047231

Supplementary Table S5-12. Differentially expressed proteins between S241 and R282 hotspot mutations

	Median			
Proteins	S241	R282	*p*	*q*
CYCLINE1	0.392395	0.90032	0.036117	0.049433
FIBRONECTIN	0.707615	-0.40426	0.049433	0.049433
MTOR	0.034511	0.38439	0.009999	0.049433
P38MAPK	0.02863	-0.34	0.034268	0.049433
PEA15	0.227595	-0.39006	0.018769	0.049433
FASN	-1.31455	0.31497	0.020997	0.049433
FOXO3APS318S321	0.22235	-0.12485	0.025143	0.049433
MYOSINIIAPS1943	0.367975	0.007241	0.024702	0.049433
P27PT198	0.17231	0.044192	0.046146	0.049433
CASPASE8	-0.14402	0.067161	0.049055	0.049433
MYOSINIIA	0.199711	-0.24695	0.035309	0.049433

Supplementary Table S5-13. Differentially expressed proteins between S241 and Y163 hotspot mutations

	Median			
Proteins	S241	Y163	*p*	*q*
BAK	-0.26836	-0.4763	0.048608	0.048661
BIM	0.44712	-0.26626	0.02083	0.039346
CHK2	-0.05354	0.33765	0.035031	0.046148
DVL3	0.458475	0.265045	0.01098	0.038848
FOXO3A	-0.01253	-0.15627	0.033394	0.046148
JNK2	-0.03965	-0.64792	0.013711	0.038848
P27	0.193045	-0.37691	0.000996	0.016397
RAD51	-0.18836	-0.058	0.035856	0.046148
FOXM1	-0.02067	1.061355	0.03771	0.046148
P27PT198	0.17231	-0.08301	0.001929	0.016397
RAB11	0.009866	-0.16609	0.004073	0.02308
ADAR1	-0.32227	0.085706	0.038004	0.046148
CDK1PY15	-0.18438	0.193455	0.016377	0.039346
BRAFPS445	-0.07772	0.10236	0.041817	0.047393
CHK1PS296	0.03269	0.140965	0.013136	0.038848
CTLA4	-0.26836	-0.4763	0.048661	0.048661
MACC1	0.44712	-0.26626	0.02083	0.039346

Supplementary Table S5-14. Differentially expressed proteins between S241 and G266 hotspot mutations

	Median			
Proteins	S241	G266	*p*	*q*
CMETPY1235	-0.05064	0.10229	0.007363	0.015453
DVL3	0.458475	0.144695	0.033508	0.033508
EGFRPY1173	-0.17609	-0.00924	0.011038	0.015453
BADPS112	0.58112	0.20335	0.000871	0.006097
PKCPANBETAIIPS660	0.28134	-0.21878	0.010619	0.015453
EPPK1	1.96035	0.058108	0.032139	0.033508
THYMIDILATESYNTHASE	-0.05064	0.10229	0.007363	0.015453

Supplementary Table S5-15. Differentially expressed proteins between C238 and R282 hotspot mutations

	Median			
Proteins	C238	R282	*p*	*q*
ERK2	0.0742	-0.63057	0.016462	0.026247
IGFBP2	2.3573	1.0886	0.019682	0.026247
PAXILLIN	0.50875	-0.1373	0.026398	0.030169
YAPPS127	1.08926	0.23633	0.016708	0.026247
FASN	-0.9988	0.31497	0.036053	0.036053
NRAS	-0.14978	0.043484	0.017724	0.026247
RICTORPT1135	0.24355	-0.01293	0.003843	0.026247
DUSP4	-0.54613	-0.1447	0.008252	0.026247

Supplementary Table S5-16. Differentially expressed proteins between C238 and Y163 hotspot mutations

	Median			
Proteins	C238	Y163	*p*	*q*
AMPKALPHA	0.230853	-0.42796	0.048328	0.049389
BCL2	-0.78954	-0.45408	0.036562	0.049389
CIAP	0.18287	0.047197	0.030348	0.049389
DVL3	0.444785	0.265045	0.039912	0.049389
EGFR	-0.30502	-0.41274	0.029068	0.049389
EGFRPY1173	-0.09896	-0.17253	0.02717	0.049389
IGFBP2	2.3573	0.77761	0.030432	0.049389
JNK2	-0.12309	-0.64792	0.016082	0.049389
STATHMIN	0.063106	-0.06878	0.049389	0.049389
RICTOR	0.330035	-0.07249	0.005529	0.049389
PKM2	0.230853	-0.42796	0.048328	0.049389
CASPASE9	-0.78954	-0.45408	0.036562	0.049389

Supplementary Table S5-17. Differentially expressed proteins between C238 and G266 hotspot mutations

	Median			
Proteins	C238	G266	*p*	*q*
AMPKALPHA	0.230853	-0.39787	0.027339	0.041203
BCL2	-0.78954	-0.37839	0.01319	0.041203
CMETPY1235	0.015889	0.10229	0.023553	0.041203
CIAP	0.18287	-0.11371	0.033218	0.041203
DVL3	0.444785	0.144695	0.036558	0.041203
IGFBP2	2.3573	1.2069	0.03826	0.041203
MEK1PS217S221	0.722445	0.105638	0.029733	0.041203
YAP	0.376975	-0.30376	0.008335	0.041203
YAPPS127	1.08926	-0.11524	0.010635	0.041203
SCD1	0.002338	0.113775	0.033853	0.041203
SETD2	-0.08249	0.093994	0.047036	0.047036
PKM2	0.230853	-0.39787	0.027339	0.041203
CASPASE9	-0.78954	-0.37839	0.01319	0.041203
THYMIDILATESYNTHASE	0.015889	0.10229	0.023553	0.041203

Supplementary Table S5-18. Differentially expressed proteins between Y220 and C163 hotspot mutations

	Median			
Proteins	Y220	Y163C	*p*	*q*
BIM	0.330215	-0.26626	0.002066	0.015594
CKIT	-0.1047	-0.51165	0.039706	0.047742
CYCLINB1	0.126899	0.94647	0.043174	0.047742
EGFR	-0.29362	-0.41274	0.012021	0.04007
EGFRPY1173	-0.03066	-0.17253	0.002599	0.015594
FOXO3A	0.046079	-0.15627	0.03722	0.047742
HSP70	0.490115	-0.35642	0.026268	0.047742
IGFBP2	2.01475	0.77761	0.044559	0.047742
KU80	-0.309	0.085044	0.010772	0.04007
MRE11	0.259445	0.144235	0.040244	0.047742
MTORPS2448	0.308105	0.40045	0.032162	0.047742
NOTCH1	-0.1198	-0.40967	0.007799	0.033424
P27	-0.05134	-0.37691	0.028776	0.047742
PR	-0.15149	-0.24385	0.037381	0.047742
STAT3PY705	0.25276	0.478415	0.049253	0.049253
STATHMIN	0.081135	-0.06878	0.015722	0.045723
BAP1C4	0.12993	0.34542	0.027178	0.047742
FASN	-0.33037	-1.35115	0.016765	0.045723
FOXM1	0.44873	1.061355	0.006025	0.030125
MYH11	0.120999	-1.01529	0.025479	0.047742
P27PT198	0.026991	-0.08301	0.038441	0.047742

PDCD4	0.72196	1.20225	0.022517	0.047742
PKCPANBETAIIPS660	0.010686	0.42061	0.034272	0.047742
RAB11	0.053138	-0.16609	0.002451	0.015594
X1433BETA	0.138875	-0.01324	0.019947	0.047742
PREX1	-0.27778	0.109561	0.04673	0.048341
MSH2	-0.03495	0.577635	0.001673	0.015594
MSH6	-0.01185	0.784535	0.043882	0.047742
MACC1	0.330215	-0.26626	0.002066	0.015594
PARPAB3	-0.1047	-0.51165	0.039706	0.047742

Supplementary Table S5-19. Differentially expressed proteins between Y220 and G266 hotspot mutations

	Median			
Proteins	Y220	G266	*p*	*q*
CMETPY1235	0.010737	0.10229	0.040581	0.040581
GAB2	1.29254	0.72878	0.015643	0.040581
THYMIDILATESYNTHASE	0.010737	0.10229	0.040581	0.040581

Supplementary Table S5-20. Differentially expressed proteins between G245 and R282 hotspot mutations

	Median			
Proteins	G245	R282	*p*	*q*
AR	-0.38258	0.81604	0.005583	0.017113
CASPASE7CLEAVEDD198	-0.12926	-0.90352	0.006582	0.017113
KU80	-0.40336	0.15987	0.040686	0.042518
MTOR	-0.19619	0.38439	0.001208	0.015704
P38MAPK	0.025807	-0.34	0.029961	0.042518
CD20	-0.1058	-0.19592	0.037535	0.042518
FASN	-0.99069	0.31497	0.040498	0.042518
TRANSGLUTAMINASE	-0.09379	-0.61802	0.016579	0.03079
ACETYLATUBULINLYS40	-0.52636	0.79168	0.03899	0.042518
ERCC1	0.100575	-0.06275	0.042518	0.042518
MYOSINIIA	0.163185	-0.24695	0.01468	0.03079
PYGBAB2	-0.38258	0.81604	0.005583	0.017113
NAPSINA	-0.12926	-0.90352	0.006582	0.017113

Supplementary Table S5-21. Differentially expressed proteins between G245 and Y163C hotspot mutations

	Median			
Proteins	G245	Y163C	*p*	*q*
ASNS	-0.02283	0.42039	0.036382	0.045625
ATM	-0.10862	1.03436	0.04121	0.045625
BAK	-0.21164	-0.4763	0.006078	0.017129
BETACATENIN	-0.23807	0.53286	0.017735	0.034362

CMETPY1235	0.095022	-0.02097	0.001445	0.010695
CHK2	-0.20692	0.33765	0.036484	0.045625
CYCLINB1	0.09756	0.94647	0.00428	0.017129
EGFR	-0.23001	-0.41274	0.008944	0.023105
EGFRPY1173	0.018276	-0.17253	0.003192	0.016492
JNK2	-0.18213	-0.64792	0.028857	0.044728
KU80	-0.40336	0.085044	0.049345	0.049345
MTOR	-0.19619	0.126169	0.026589	0.044728
MTORPS2448	0.08185	0.40045	0.000999	0.010695
NOTCH1	0.008695	-0.40967	0.004923	0.017129
S6PS235S236	-0.20937	0.71961	0.038366	0.045625
S6PS240S244	-0.19805	0.54102	0.026012	0.044728
CD20	-0.1058	-0.18073	0.047278	0.048854
FOXM1	0.173269	1.061355	0.001725	0.010695
PDCD4	0.44393	1.20225	0.03965	0.045625
PKCPANBETAIIPS660	-0.19937	0.42061	0.043907	0.046935
ERCC1	0.100575	-0.15622	0.013175	0.029173
MSH2	-0.965	0.577635	0.001081	0.010695
MSH6	0.058597	0.784535	0.028852	0.044728
CDK1PY15	-0.11739	0.193455	0.009944	0.023713
CHK1PS296	0.053112	0.140965	0.005177	0.017129
PYGL	-0.02283	0.42039	0.036382	0.045625
PYGM	-0.10862	1.03436	0.04121	0.045625
CTLA4	-0.21164	-0.4763	0.006078	0.017129
KEAP1	-0.23807	0.53286	0.017735	0.034362
MACC1	0.225345	-0.26626	0.0379	0.045625
THYMIDILATESYNTHASE	0.095022	-0.02097	0.001445	0.010695

Supplementary Table S5-22. Differentially expressed proteins between G245 and G266 hotspot mutations

	Median			
Proteins	G245	G266	*p*	*q*
SHCPY317	0.24244	0.105554	0.019198	0.045465
X4EBP1PT70	-0.23119	0.036577	0.043175	0.045465
ACVRL1	0.106505	-0.0721	0.03391	0.045465
ANNEXIN1	0.212075	-0.45296	0.025922	0.045465
JAB1	0.01228	-0.16196	0.037992	0.045465
ERCC1	0.100575	-0.06561	0.045465	0.045465

Supplementary Table S5-23. Differentially expressed proteins between R248 and Y163C hotspot mutations

	Median			
Proteins	R248	Y163C	*p*	*q*
BIM	0.49265	-0.26626	0.002162	0.015115
CMETPY1235	0.02811	-0.02097	0.009454	0.020436
CYCLINB1	0.12292	0.94647	0.00047	0.0094
EGFR	-0.25826	-0.41274	7.05E-05	0.002282
EGFRPY1173	-0.08996	-0.17253	0.003023	0.015115
FOXO3A	0.010265	-0.15626	0.005322	0.016936
GSK3ALPHABETAPS21S9	-0.5077	0.040349	0.019902	0.031843
HSP70	0.24339	-0.35642	0.015551	0.027969
IRS1	-0.21036	-0.34309	0.042644	0.04734
JNK2	-0.35363	-0.64792	0.0472	0.04734
MAPKPT202Y204	0.57599	1.2262	0.002477	0.015115
MEK1PS217S221	0.058121	0.51562	0.027606	0.036877
MTORPS2448	0.22987	0.40045	0.005252	0.016936
P27	0.089742	-0.37691	0.000717	0.00956
P90RSKPT359S363	0.11243	0.482535	0.024909	0.036875
PEA15	-0.0409	-0.23836	0.044331	0.04734
PR	-0.20354	-0.24385	0.009707	0.020436
RAD51	-0.12785	-0.058	0.015307	0.027969
STATHMIN	0.014436	-0.06878	0.016082	0.027969
SYK	0.53168	1.2806	0.027593	0.036875
BADPS112	0.29972	0.62223	0.002893	0.015115
CD20	-0.1281	-0.18073	0.042775	0.04734
FASN	-0.92809	-1.35115	0.030844	0.03856
FOXM1	0.27395	1.061355	0.005893	0.016936
P27PT198	0.023803	-0.08301	0.027656	0.036875
PDCD4	0.62285	1.20225	0.022032	0.033896
PKCPANBETAIIPS660	0.073803	0.42061	0.045139	0.04734
RAB11	-0.03399	-0.16609	0.006351	0.016936
RICTOR	0.34214	-0.07249	0.005707	0.016936
X1433BETA	0.094388	-0.01324	0.04734	0.04734
MSH2	0.26744	0.577635	0.019799	0.031843
CDK1PY15	-0.06646	0.193455	0.005478	0.016936
BRAFPS445	-0.03765	0.10236	0.028966	0.037375
CD26	-0.07333	-0.18406	0.043406	0.04734
CHK1PS296	0.058095	0.140965	0.006094	0.016936
ERCC5	-0.1612	0.206275	0.034829	0.04734
P16INK4A	1.0044	1.65385	0.012769	0.020358
CTLA4	-0.26468	-0.4763	0.009654	0.020358
MACC1	0.49265	-0.26626	0.002162	0.015115

THYMIDILATESYNTHASE	0.02811	-0.02097	0.009454	0.020436

Supplementary Table S5-24. Differentially expressed proteins between R248 and G266 hotspot mutations

	Median			
Proteins	R248	G266	*p*	*q*
CMETPY1235	0.02811	0.10229	0.02417	0.48726
NOTCH1	-0.27195	0.132825	0.009789	0.48726
SHCPY317	0.19621	0.105554	0.012788	0.48726
YAP	0.1334	-0.30376	0.040687	0.48726
YAPPS127	0.91375	-0.11524	0.044595	0.48726
PDCD4	0.62285	0.47756	0.048726	0.48726
EPPK1	2.1611	0.058108	0.037959	0.48726
ACVRL1	0.075018	-0.0721	0.034278	0.48726
ANNEXIN1	0.06494	-0.45296	0.036956	0.48726
THYMIDILATESYNTHASE	0.02811	0.10229	0.02417	0.48726

Supplementary Table S5-25. Differentially expressed proteins between H179 and Y163C hotspot mutations

	Median			
Proteins	H179	Y163C	*p*	*q*
AR	0.5937	-0.12727	0.038545	0.044051
BAK	-0.32657	-0.4763	0.032467	0.041011
BIM	0.90307	-0.26626	0.022439	0.034961
CMETPY1235	0.029756	-0.02097	0.044165	0.046085
CDK1	-0.25127	0.107683	0.013604	0.027208
EGFR	-0.16522	-0.41274	0.009279	0.027208
EGFRPY1173	0.061359	-0.17253	0.015925	0.0294
MEK1PS217S221	-0.29033	0.51562	0.010021	0.027208
P27	0.1004	-0.37691	0.004737	0.027208
P90RSKPT359S363	0.21218	0.482535	0.046573	0.046573
CD20	-0.11195	-0.18073	0.026565	0.037504
FASN	-0.37009	-1.35115	0.004861	0.027208
FOXM1	-0.04458	1.061355	0.002095	0.027208
PDCD4	0.63423	1.20225	0.012384	0.027208
PEA15PS116	0.18886	-0.09051	0.037672	0.044051
PKCPANBETAIIPS660	-0.35694	0.42061	0.023307	0.034961
X1433BETA	0.077331	-0.01324	0.010102	0.027208
MSH2	0.17576	0.577635	0.012643	0.027208
CDK1PY15	-0.32891	0.193455	0.004475	0.027208
CHK1PS296	-0.01989	0.140965	0.008119	0.027208
CTLA4	-0.32657	-0.4763	0.032467	0.041011
MACC1	0.90307	-0.26626	0.022439	0.034961

THYMIDILATESYNTHASE	0.029756	-0.02097	0.044165	0.046085
ALPHACATENIN	-0.25127	0.107683	0.013604	0.027208

Supplementary Table S5-26. Differentially expressed proteins between H179 and G266 hotspot mutations

	Median			
Proteins	H179	G266	*p*	*q*
CD49B	-0.15721	-0.32079	0.04625	0.04625
NOTCH1	-0.27173	0.132825	0.031524	0.04625
SMAD4	0.010692	-0.06832	0.010117	0.04625
EPPK1	1.9489	0.058108	0.036422	0.04625
CASPASE3	-0.08046	-0.28902	0.032372	0.04625
SYNAPTOPHYSIN	-0.15721	-0.32079	0.04625	0.04625

Supplementary Table S5-27. Differentially expressed proteins between H193 and Y163C hotspot mutations

	Median			
Proteins	H193	Y163C	*p*	*q*
AMPKALPHAPT172	0.52521	-0.54382	0.045286	0.047691
CYCLINE1	1.178	0.30059	0.015103	0.047691
GSK3ALPHABETA	0.089741	-0.11162	0.028358	0.047691
MAPKPT202Y204	-0.33694	1.2262	0.023508	0.047691
MEK1PS217S221	-0.341	0.51562	0.018556	0.047691
MTORPS2448	0.14364	0.40045	0.00846	0.047691
P70S6KPT389	-0.11114	0.35094	0.038435	0.047691
YB1PS102	-0.1137	0.20653	0.030319	0.047691
BADPS112	-0.19014	0.62223	0.024758	0.047691
CD20	-0.12264	-0.18073	0.043008	0.047691
P27PT198	0.031505	-0.08301	0.013208	0.047691
PKCPANBETAIIPS660	-0.34803	0.42061	0.011544	0.047691
EPPK1	-0.13753	2.03085	0.026281	0.047691
PREX1	-0.33875	0.109561	0.033556	0.047691
ERCC1	-0.00016	-0.15622	0.046365	0.047691
CDK1PY15	-0.17412	0.193455	0.00317	0.030115
CHK1PS296	0.008254	0.140965	0.001667	0.030115
SHP2PY542	-0.30073	0.091871	0.047691	0.047691
PYGB	0.52521	-0.54382	0.045286	0.047691

Supplementary Table S5-28. Differentially expressed proteins between H193 and G266 hotspot mutations

	Median			
Proteins	H193	G266	*p*	*q*
CYCLIND1	-0.13621	0.074247	0.024893	0.03384

CYCLINE1	1.178	0.24591	0.005254	0.03384
HSP70	-0.16324	0.49093	0.034687	0.038616
MAPKPT202Y204	-0.33694	0.405115	0.038616	0.038616
P70S6KPT389	-0.11114	0.52465	0.013196	0.03384
YB1PS102	-0.1137	0.070812	0.02538	0.03384
ARAFPS299	-0.01151	0.17907	0.010422	0.03384
JAB1	0.072759	-0.16196	0.018741	0.03384

Supplementary Table S5-29. Differentially expressed proteins between G275 and Y163C hotspot mutations

	Median			
Proteins	G275	Y163C	*p*	*q*
BIM	0.567545	-0.26626	0.001212	0.010908
CRAF	-0.09872	0.053441	0.041347	0.043779
EGFR	-0.15385	-0.41274	0.002025	0.01215
MRE11	0.29776	0.144235	0.02785	0.035807
NOTCH1	-0.13875	-0.40967	0.012649	0.020698
P27	0.320785	-0.37691	0.009917	0.020698
PEA15	0.38933	-0.23836	0.011279	0.020698
SYK	0.13573	1.2806	0.020158	0.030237
BAP1C4	0.097007	0.34542	0.01246	0.020698
FOXM1	0.100945	1.061355	0.006535	0.016804
P27PT198	0.105484	-0.08301	0.005269	0.015807
PDCD4	0.47762	1.20225	0.044784	0.044784
PDK1	-0.10566	0.032754	0.023672	0.032777
CDK1PY15	-0.14936	0.193455	0.004134	0.014882
BCL2A1	0.344535	0.029144	0.039373	0.043779
CHK1PS296	0.027779	0.140965	0.003369	0.014882
CTLA4	-0.21414	-0.4763	0.034453	0.041344
MACC1	0.567545	-0.26626	0.001212	0.010908

Supplementary Table S5-30. Differentially expressed proteins between G275 and G266 hotspot mutations

	Median		
Proteins	G275	G266	*p*
CYCLINE1	1.117055	0.24591	0.033095

Supplementary Table S5-31. Differentially expressed proteins between Y234 and Y163C hotspot mutations

	Median			
Proteins	Y234	Y163C	*p*	*q*
CJUNPS73	-0.0384	0.34139	0.019647	0.037067
CMETPY1235	0.066738	-0.02097	0.044365	0.045935
CYCLINB1	-0.05087	0.94647	0.004928	0.037067
EGFR	-0.08406	-0.41274	0.023055	0.037067
EGFRPY1068	-0.32234	0.037959	0.041258	0.045935
HER3PY1289	-0.17101	-0.04832	0.045935	0.045935
JNK2	-0.07367	-0.64792	0.011954	0.037067
KU80	-0.2707	0.085044	0.011873	0.037067
FOXM1	-0.01408	1.061355	0.001622	0.025952
RICTOR	0.59617	-0.07249	0.030524	0.044399
CDK1PY15	-0.19901	0.193455	0.014908	0.037067
CD26	-0.09618	-0.18406	0.023167	0.037067
CHK1PS296	0.009367	0.140965	0.015566	0.037067
SHP2PY542	-0.26126	0.091871	0.043409	0.045935
NRF2	-0.0384	0.34139	0.019647	0.037067
THYMIDILATESYNTHASE	0.066738	-0.02097	0.044365	0.045935

Supplementary Table S5-32. Differentially expressed proteins between Y234 and G266 hotspot mutations

	Median			
Proteins	Y234	G266	*p*	*q*
EGFRPY1068	-0.32234	-0.09865	0.048485	0.048485
IGFBP2	0.42483	1.2069	0.037945	0.045534
PAI1	-0.51274	0.14914	0.024481	0.037674
RAD51	-0.23173	-0.00242	0.025116	0.037674
STAT5ALPHA	0.74527	0.201825	0.02331	0.037674
ACVRL1	0.16479	-0.0721	0.011781	0.037674
